# Integrating Microbiomes for Regenerative Food Systems: Recent Insights, Implementations, and Emerging Trends

**DOI:** 10.1002/fsn3.71312

**Published:** 2025-12-02

**Authors:** Muhammad Tayyab Arshad, Sammra Maqsood, Md. Sakhawot Hossain, Farhang Hameed Awlqadr, Abdul Rauf, Iffat Ullah, Ali Ikram, Ayesha Bibi, Sayeed Mukhtar, Muhammed Adem Abdullahi

**Affiliations:** ^1^ Functional Food and Nutrition Program, Center of Excellence in Functional Foods and Gastronomy, Faculty of Agro‐Industry Prince of Songkla University Hat Yai Songkhla Thailand; ^2^ National Institute of Food Science and Technology University of Agriculture Faisalabad Faisalabad Pakistan; ^3^ Department of Nutrition and Food Technology Jashore University of Science and Technology Jasho Bangladesh; ^4^ Department of Nutrition and Food Engineering Daffodil International University Dhaka Bangladesh; ^5^ Food Science and Quality Control, Halabja Technical College Sulaimani Polytechnic University Sulaymaniyah Iraq; ^6^ Department of Pharmaceutical Chemistry, Faculty of Pharmaceutical Sciences Prince of Songkla University Hat Yai Songkhla Thailand; ^7^ Drug Delivery System Excellence Center (DDSEC) Pharmaceutical Sciences Prince of Songkla University Hat‐Yai Songkhla Thailand; ^8^ University Institute of Food Science and Technology The University of Lahore Lahore Pakistan; ^9^ Department of Human Nutrition Women University Mardan Khyber Pakhtunkhwa Pakistan; ^10^ Organic and Medicinal Chemistry Research Lab, Department of Chemistry, Faculty of Science University of Tabuk Tabuk Saudi Arabia; ^11^ Department of Food Science and Postharvest Technology, Jimma University College of Agriculture and Veterinary Medicine Jimma University Jimma Ethiopia

**Keywords:** biofertilizers, fermentation, machine learning, sustainable agricultural practices

## Abstract

Microbiomes play a central role in food science by influencing food quality, safety, sustainability, and human health. This review brings together contemporary advancements in the understanding and use of microbiomes in the food system, with a focus on sustainable agriculture, fermentation, food safety, and nutrition. We discuss both traditional approaches (e.g., natural fermentation and soil management) and new high‐tech strategies, including precision microbiome engineering, synthetic biology, and machine learning. The major applications under consideration are microbiome‐based food preservation, biofertilisers and biopesticides, waste valorisation, and alternative protein production. The socioeconomic context is also being considered, recognizing that while recent advances are more pragmatic in high‐income countries, traditional and low‐input approaches are still critical in low‐ and middle‐income country (LMIC) settings. The emphasis in this review is laid on food science and food systems, and nutrition and health effects are considered as downstream consequences. The readership includes food scientists, microbiologists, agricultural researchers, and policy decision‐makers who care about the integration of microbiome science into resilient and sustainable food systems. By presenting a fair representation of standard and advanced microbiome techniques, this review delineates the prospects and challenges in the application of microbial communities in shaping the future of food.

## Graphical abstract

This review explores how the specific understanding of native microbiome dynamics can aid in advancing agricultural practices and food safety. It also reviews recent advances in microbiome‐targeted technologies, including personalized nutrition, functional foods, and engineered approaches. Further exploration is needed into how microbiomes could be utilized in agricultural systems to make them more sustainable under microbe‐present or adverse conditions.

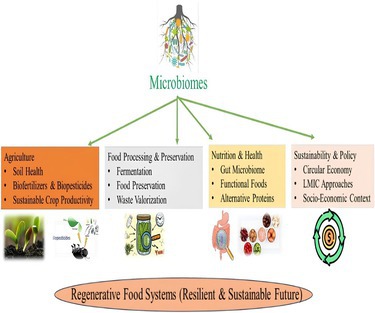

## 1 Introduction

The concept of microbiome has revolutionized food science by introducing new ways in which microbial interactions can be examined across time scales and among different components within a food ecosystem (Aamir et al. 2021). For conceptual clarity, this review positions food science as its central domain, situates food systems as the applied framework for sustainability, and addresses nutrition and health as its downstream implications. The microbiome comprises various communities of microorganisms, viruses, fungi, bacteria, and archaea that inhabit a particular environment such as production systems or food matrices (Abu‐Thabit et al. 2022). Food science in this context includes food composition, processing, safety, and quality science and its interfaces with agriculture (primary production systems), nutrition, and health (consumer endpoints). The food environments used in this review are the microbial and ecological environments of food production, processing, and storage systems, and not the sociocultural sense that is sometimes applied to public health. Accordingly, the term food environment is consistently used to denote microbial and ecological contexts rather than sociocultural ones (Albuquerque et al. 2022). A microbiome is thus defined by its emphasis on entire microbial communities and their ecological interactions, in contrast to the more reductionist strategies of mainstream microbial science, which are more centered on single species or strains in isolation. Such microbiome‐based strategies thus provide a system‐level understanding to complement and build on typical microbial approaches (Zhu et al. 2023).

At the center of sustainable food systems, the microbiome plays a key role in the production and storage of bacteria in agriculture, food production, and consumer safety (Bakker et al. 2020). Subsequent research has sought to shape these microbial communities with the aim of supporting resilient and sustainable food systems, whereas prior studies have focused on characterizing the microbes detected in foods (Afridi et al. 2022; Alcock et al. 2021). The move from descriptive microbiome studies to precision engineering of the gut in lessons such as this one highlights how critical understanding these complex microbial communities has become if we hope to address some of our most pressing food quality and safety issues for a sustainable future (Areniello et al. 2023). For example, microbiological therapies can be developed to enhance nutritional profiles, reduce food spoilage, and decrease the environmental burden associated with farming practices (Astapati and Nath 2023). This review serves three purposes: (1) to provide an overview of the function of microbiomes in food science, and (2) to examine a brief timeline of research conducted on microbiomes and their outcomes for food sustainability. Evidence investigates how we can develop precisely tailored microbiome networks designed to enhance interspecies ecological interactions between taxa in support of sustainable agriculture to optimize the system and make it resilient (Ballerstedt et al. 2021). This is because the study of natural fermentation processes, in which microbial populations were first recognized for their role in food modification and preservation (Bakker et al. 2020), has also initiated microbiome research. To date, these studies have primarily focused on determining which species are the most dominant players in other foods (such as cheese and yogurt). When new molecular biology techniques, metagenomics, and next‐generation sequencing are available, it becomes possible for scientists to begin to unveil the vast diversity of food‐associated microbiomes (Balkir et al. 2021). This is possible with these new technologies, which will help scientists study more complex ecological webs underneath food systems instead of just counting types (Ballerstedt et al. 2021). Food science applications in the field of microbiome research have been growing, with some impacts on various existing food science applications. For many essential processes, such as managing microbiomes in exact ways to improve plant health and yield (Bao et al. 2023), regulating microbial interactions is a necessary aspect of operations. By engineering the plant microbiome, scientists can promote beneficial interactions to enhance the growth and nutrient uptake of plants and protect them against pathogens (Beattie et al. 2024). Similar to higher organisms, the microbiome is a critical element for food quality and safety. Microbial contamination is one of the most common causes of foodborne illness (Beck et al. 2021), and it is vital to track and control microbial communities in environments related to food production, thus ensuring their safety. Sophisticated sequencing methods have been developed and implemented to identify harmful microbes at the strain level in real time, which can help prevent contamination and enhance quality control (Ferone et al. 2020). At the same time, there is a growing appetite for using microbial communities to boost the nutrition of what we eat. For example, the gut microbiota not only facilitates digestion and nutrient absorption, but also plays critical roles in immune function, host metabolism, neurologic signaling through the gut–brain axis, and xenobiotic metabolism (Berg et al. 2020).

Research on diet and gut microbiota provides the possibility of opening new avenues for functional foods implicated in boosting digestive health or reducing the risk of developing chronic diseases (Hossain, Wazed, Shuvo, et al. 2025). This also represents a line of research on the potential use of recycling waste into valuable products to support more sustainable food systems (Carmody and Bisanz 2023). Microbial consortia also convert food waste into biofuels and bioplastics to reduce the environmental footprint of the food industry (Catania et al. 2021). One of the major contributions of microbiome research is its support for sustainable food systems, which is well appreciated in food science (Compant et al. 2024). They also affect agricultural practices by improving crop tolerance to environmental conditions and by enhancing soil quality. The use of plant microbiomes has been shown to reduce ecological pollution by augmenting soil nutrient availability, reducing fertilizer application, and controlling soil pathogens, which are key to sustainable farming (Siddiqui et al. 2025). Microbial inoculants, also called biofertilizers or biostimulants, are being developed to increase favorable microbial relationships in the rhizosphere, thus improving agricultural productivity and sustainability (De Filippis et al. 2021). Valorization of side‐streams is emerging as a new method whereby microbiomes can be used to convert food processing waste into biofuels, bioplastics, and microbial proteins.

To decompose plastics and convert agricultural production waste streams into bio‐based products such as second‐generation microbial proteins or bioplastics, scientists are genetically engineering microbial consortia to biodegrade plastics and convert agri‐side‐streams into bio‐based products. These engineered products can then be adopted by farmers and industry stakeholders (De Vrieze et al. 2018).

These innovations are consistent with the growing needs of the food sector, where circular economies are embraced to reduce waste and optimize resource recycling (Dey and Ray Chaudhuri 2023). The utilization of microbiomes allows for the conversion of food waste into valuable products and helps mitigate the negative impacts of food production and consumption on the environment (Dubey and Kumar 2022). In addition, the integration of microbiomes into precision farming may completely reshape food production systems. It is effective in manipulating specific microbial communities through precise microbiome management for optimizing crops under specific climatic conditions (Eleftheriadou et al. 2017). This plan is crucial in view of climate change because it increases the stress already present in conventional agricultural methods owing to variable climatic conditions and declining resources (El‐Metwally et al. 2023). With the aid of microbiomes, scientists can build resilient food systems that can withstand environmental shocks and remain productive (French et al. 2021).

This review aims to shed light on the importance of microbial communities in food safety and the efficiency of food production via complex dynamics. It also examines high‐tech approaches in microbiome engineering and how these approaches may be applied to challenges such as resource optimization, environmental impact, and food security. This review provides an in‐depth perspective on the integration of microbiome science into the development of resilient and sustainable food systems. To provide a brief overview, this review first discusses microbiome applications in food quality and safety, subsequently highlighting their applications in fermentation and biopreservation, sustainable agriculture (biopesticides and biofertilizers), waste valorization and alternative protein generation, tailored consumer well‐being and nutrition, and finally innovative multi‐omics tools, machine learning, and regulatory considerations. In the same manner, this review recognizes the merits of low‐tech and ancient microbiome practices, such as natural fermentation, indigenous soil fertility management, and diet–lifestyle interactions with gut microbiomes. These perspectives are particularly important in low‐ and middle‐income country (LMIC) settings, where resource availability and cultural expectations frame what is feasible compared with high‐income, industrialized food systems.

## 2 Applications of Microbiomes in Food Safety and Quality

Microbial consortia are essential for improving the quality and safety of food. Microbiomes are observed within the food industry as factors contributing to food hygiene and shelf life, which are areas of concern for both manufacturers and end users (Ballerstedt et al. 2021). Foodomics is a newly emerging area within this field of research that addresses food safety and quality issues by integrating modern technologies with microbiome analyses (Frey‐Klett et al. 2011). One can also study the microbiomes of food production facilities to investigate the sources of possible contamination and take preventive measures to ensure food safety (García‐Depraect et al. 2021). This approach has already been adopted in the dairy and meat sectors, where microbiome surveillance helps to increase the shelf life and microbiological safety of products (Ghannam and Techtmann 2021). The utilization of microbiomes in food incorporates another aspect: the development of functional foods with health benefits. Probiotics, prebiotics, and synbiotics are intended for nutrition to modulate the gut microbiota to improve digestion and eventually prevent chronic diseases (Ghannoum et al. 2021). It is necessary to distinguish between single‐strain applications, such as the use of *Lactobacillus* in yogurt or *Saccharomyces* in bread fermentation, and microbiome‐scale consortia with many microbial species that interact to shape food quality, safety, and ecological resilience.

With growing awareness of the interplay between the diet, microbiome, and health aspects, functional foods are on the rise (González et al. 2021). At the same time, these products represent a promising approach to the treatment of dietary‐related health (e.g., obesity, diabetes, and cardiovascular disease, as they were developed based on microbiome research (Gopal and Gupta 2019)). This perspective reflects the evolution of food science microbiome research, from fundamental studies of microbial diversity to fast‐growing applications in precision engineering and sustainability. Microbiomes can also provide new solutions to old problems plaguing food systems, and they are required to innovate in the future for safer, more nutritious foods. After studying microbiomes, professors can use ecological interactions to design new microbe‐related waste removal applications, food safety products, and sustainable agricultural practices (Astapati and Nath 2023). Continuous microbiome research will allow more robust and sustainable global food systems to serve a growing population (Abu‐Thabit et al. 2022).

## 3 Microbiome Ecology in Food Environments

### 3.1 Ecological Interactions in Food Microbiomes

The microbiomes of food environments are structured webs of microbial species that participate in mutualistic, competitive, and codependent interactions. These interactions are involved in determining microbial makeup, which will also impact food preservation/quality and safety. For instance, mutualistic interactions between bacteria and yeasts, which are common in fermented foods, help enhance the nutritional quality of food while imparting distinct levels to its taste. Bread fermentation provides an example: the leavening of bread through sourdough fermentation illustrates a cooperative interaction between yeast and lactic acid bacteria because all microbes study outputs, that is, yeast and lactic acid bacteria (LAB), clearly coordinate by helping each other with the rise or profile determinants, as well as metabolic benefits (Gopal and Gupta 2019). Filterfasta full Download ×Fasta Post XML Consortility? However, this interaction provides an opportunity to think more about the complex layering of microbes required for critical transformation in food (Astapati and Nath 2023; Compant et al. 2024; Frey‐Klett et al. 2011; Kern et al. 2021; Lee et al. 2022). Interestingly, in food‐related contexts, commensal relationships (in which one microbial species benefits the other without significantly hurting it) are also abundant. For instance, certain *Lactobacillus* species are part of the normal flora of the mouth, gut, and vagina by fermenting milk to make yogurt (Kern et al. 2021). These links contribute to the quality of food, although not too much distance from providing environmental balance for bacteria (Karabulut et al. 2021).

In contrast, antagonistic activities have become important for regulating microbial populations in food systems. The latter refers to the fact that microorganisms use different antimicrobial compounds (e.g., nitric acid and bacteriocins) to outcompete or kill rival bacterial organisms such as pathogens. Lactic acid bacteria (LAB) are commonly used in fermented foods that produce lactic acid, which contributes to pH reduction and inhibits the growth of undesirable microorganisms such as *Listeria monocytogenes* or spoilage organisms (González et al. 2021; Karabulut et al. 2021). Because of these antagonistic actions that impede the growth of pathogenic bacteria, they are environmentally friendly control methods for food preservation and thus contribute to biopreservation (Table 1) (González et al. 2021).

**TABLE 1:** Ecological interactions in food microbiomes.

Type of interaction	Description	Examples	References
Mutualism	A relationship where both microbial species benefit	Fermentative microbes producing organic acids while other species utilize these acids for further breakdown, enhancing flavor	(Bakker et al. 2020; Hossain, Wazed, Shuvo, et al. 2025)
Commensalism	One species benefits without affecting the other	Yeasts producing growth factors that stimulate the growth of lactic acid bacteria in fermented products	(Areniello et al. 2023; Eleftheriadou et al. 2017)
Parasitism	One species benefit at the expense of another	Pathogenic bacteria such as *Listeria monocytogenes* exploiting nutrient resources in food while harming host microbiota	(Frey‐Klett et al. 2011; Infante‐Villamil et al. 2021)
Competition	Microorganisms compete for limited resources, affecting their survival and growth	Mold and bacteria competing for simple sugars in bread	(Compant et al. 2024; Dubey and Kumar 2022)
Amensalism	One species inhibits another without benefiting itself	Production of antimicrobial peptides by certain bacteria that inhibit spoilage organisms	(Beattie et al. 2024; Dey and Ray Chaudhuri 2023)
Predation	One organism preys upon another	Protozoa consuming bacteria in biofilms on food surfaces	(Carmody and Bisanz 2023; Karpe et al. 2023)
Syntrophy	Cooperative interaction where one species relies on the byproducts of another for survival	Methanogens utilizing hydrogen and carbon dioxide produced by fermentative bacteria in cheese production	(Ballerstedt et al. 2021; De Filippis et al. 2021)
Neutralism	Coexistence without any significant interaction or effect on each other	Co‐occurrence of diverse bacterial species in stored food environments with no apparent interaction	(Gregory et al. 2023; Hossain et al. 2024)
Facilitation	One species modifies the environment, benefiting others	*Lactobacillus* reducing pH, creating favorable conditions for yeast growth in sourdough	(Afridi et al. 2022; Astapati and Nath 2023)
Quorum sensing	Communication among microbial populations via chemical signals for coordinated activity	Biofilm formation on food‐processing equipment by *Pseudomonas aeruginosa*	(Beck et al. 2021; Hernández Medina et al. 2022)
Biofilm formation	Cooperative microbial interactions leading to structured communities	Mixed biofilms of *Listeria* and *Escherichia coli* on dairy equipment	(Bao et al. 2023; Hussain et al. 2023)
Cross‐feeding	Exchange of metabolites between species	*Saccharomyces cerevisiae* producing ethanol utilized by acetic acid bacteria in vinegar fermentation	(Berg et al. 2020; Ghannoum et al. 2021)
Antagonism	One microorganism inhibits the growth of another	Use of bacteriocins by starter cultures to inhibit spoilage bacteria in dairy products	(Catania et al. 2021; García‐Depraect et al. 2021)
Co‐evolution	Evolution of microorganisms due to long‐term interactions	Adaptation of probiotics to human gut microbiomes in fermented foods	(Abu‐Thabit et al. 2022; Ghannam and Techtmann 2021)
Symbiosis	Close and long‐term biological interaction	Yeast‐bacteria consortia in kombucha production	(Aamir et al. 2021; Balkir et al. 2021)
Pathogenesis	Microbial species causing disease in humans or spoilage in food	*Salmonella* growth in contaminated poultry	(Ferone et al. 2020; González et al. 2021)
Decomposition	Microbial breakdown of organic material into simpler compounds	Degradation of food proteins by proteolytic bacteria during spoilage	(French et al. 2021; Herrero et al. 2021)
Horizontal gene transfer	Genetic material exchange among microbes, aiding adaptation	Antibiotic resistance transfer among bacteria in raw meats	(De Vrieze et al. 2018; Hossain, Wazed, et al. 2025)
Epiphytism	Microorganisms living on the surface of plants or food without harm	Epiphytic bacteria on fresh produce	(Alcock et al. 2021; Karabulut et al. 2021)
Endosymbiosis	Microbes living inside cells of another organism, providing mutual benefits	Nitrogen‐fixing bacteria in legumes contributing to soil microbiomes.	(Compant et al. 2024; Jansson et al. 2023)
Fermentation	Metabolic processes converting sugars into acids, gases, or alcohol	Conversion of lactose to lactic acid by *Lactobacillus* in yogurt	(Dey and Ray Chaudhuri 2023; Gopal and Gupta 2019)
Spoilage	Uncontrolled microbial activity leading to food deterioration	Fungal growth in stored grains causing aflatoxin production	(Astapati and Nath 2023; Graw et al. 2021)
Acidogenesis	Production of acids by microbes impacting the food environment	Lactic acid bacteria lowering pH during sauerkraut fermentation	(Balkir et al. 2021; El‐Metwally et al. 2023)
Biocontrol	Use of beneficial microbes to inhibit pathogens or spoilage organisms	*Bacillus subtilis* applications to reduce mold on fruits	(Siddiqui et al. 2025; Kabir et al. 2024)
Microbial succession	Sequential growth of microbial communities	Development of specific microbial profiles during wine fermentation	(Carmody and Bisanz 2023; Kaul et al. 2021)
Stress tolerance	Adaptations allowing microbes to survive food processing conditions	Heat‐resistant spores of *Clostridium botulinum* in canned foods	(Ballerstedt et al. 2021; Kaul et al. 2017)

### 3.2 Impact of Environmental Factors on Microbial Communities

In food settings, temperature, pH, water activity, and other factors strongly influence the microbial population dynamics. Temperature: One of the key factors affecting microbial activity and growth is temperature. An example of this is temperature control during fermentation operations, which would allow the activation of beneficial bacteria growth while preventing the formation of rotting organisms (Hernández Medina et al. 2022). Such low temperatures can favor the growth of beneficial bacteria such as *L. lactis* , which is often used in dairy fermentation. However, high temperatures may provide suitable conditions for spoilage bacteria to grow (Infante‐Villamil et al. 2021). The types of bacteria that thrive in food products are significantly affected by pH levels. Lactic acid bacteria (LAB) tend to grow well in acidic environments, such as those found in fermented vegetables or dairy products. These bacteria lower pH levels by producing organic acids, which play a crucial role in food preservation (Graw et al. 2021). This process is vital for ensuring the safety and longevity of food by inhibiting the growth of spoilage organisms such as molds and pathogens (González et al. 2021). An essential aspect of food microbiomes is the water activity, which measures the amount of free water available for microbial growth. Microorganisms require specific levels of water activity to thrive; therefore, food preservation methods, such as drying, salting, or adding sugars, are employed to lower water activity. Foods with low water activity, such as salted fish or dried fruits, exhibit reduced microbial activity, which helps to prevent spoilage and extend their shelf life. These environmental controls enable the modification of food microbiomes, fostering the growth of the desired microbial communities while inhibiting unwanted organisms (González et al. 2021).

### 3.3 Role of Microbiomes in Food Fermentation and Biopreservation

Fermentation is an excellent method for food production and has been used since the very beginning. It is a process that is boosted by microbiomes and changes the metabolic process so that nutritional value is improved, flavor is better, and there is a rich and creamy consistency. Food ingredient commensal communities that ferment food molecules comprise the community during the process of food fermentation, which is the case for food batters, such as cheese, yogurt, and sauerkraut. Different methods may result in their presence. For instance, LAB make an outstanding contribution to dairy fermentation by converting lactose into lactic acid, which in turn lowers the pH and forms milk proteins that coagulate into curds (Hussain et al. 2023). A similar process is the one which sees *Saccharomyces cerevisiae* yeasts use sugar for fermentation, resulting in alcohol production and the formation of carbon dioxide that not only gives taste but also causes the dough to rise, thus resulting in bread and beer fermentation (Kern et al. 2021). The use of microbiomes has grown owing to advanced fermentation methods. These methods select specific microbial strains and tweak their metabolism to boost the process and improve food quality. Progress in synthetic biology allows us to create groups of microbes that ferment more. This leads to a more consistent and pleasing flavor, texture, and safety outcome (Gregory et al. 2023; Kovac et al. 2017). Scientists are now understanding how microbes interact during fermentation. They can be used to adjust the conditions to obtain specific results, such as better probiotic benefits or the creation of bioactive compounds. To do this, they use multi‐omics approaches that combine genomics, transcriptomics, and metabolomics (Graw et al. 2021). These include fermented plant‐based foods that look and taste like regular meat and dairy items. These breakthroughs not only satisfy customers who want plant‐based options but also help the environment by reducing the need for animal‐sourced materials (González et al. 2021; Jansson et al. 2023). Traditional fermentation methods such as sourdough, kimchi, sauerkraut, tempeh, and yogurt demonstrate how microbial consortia have been managed by humans for decades without advanced technologies. These low‐cost, locally embedded methods remain at the center of many LMIC food systems, producing safe, high‐quality foods and demonstrating that microbiome‐based food innovation is not necessarily high‐tech (Valentino et al. 2024).

### 3.4 Biopreservation Potential

Biopreservation extends the shelf life of food products by using natural microbiomes and their metabolites, instead of artificial preservatives. This method uses the antibacterial properties of specific microbial species, such as LAB, to prevent pathogenic organisms and spoilage in various foods. LAB produce hydrogen peroxide, bacteriocins, and organic acids, which inhibit foodborne pathogens (Hernández Medina et al. 2022). Research shows that adding LAB to meat and dairy products can prevent the growth of harmful bacteria, such as Salmonella and Listeria, while maintaining the sensory appeal of the food (Ke et al. 2021). As a result, it enhances food safety while simultaneously fulfilling consumers' quest for natural ways of preserving foods. For example, the bacteriocins *Lactococcus lactis* and nisin have been used in the biopreservation of cheese because they inhibit the spoiling of cheese without altering its quality (Hussain et al. 2023). Biopreservation does not harm sustainable food production needs and thus contributes to the development of cleaner‐label foods through the reduction in chemical preservatives used. The potential combinability of microbiomes with other conservative methods, such as the use of olfactory agents, plant extracts, or probiotics, to strengthen the actions of natural preservatives on food safety and storability, has been considered in recent research. For instance, it has been further proven that the incorporation of LAB and rosemary extract can help control microbial growth in fresh‐cut vegetables, giving them a longer shelf life (Gopal and Gupta 2019).

Furthermore, there are other interesting and emerging possibilities for enhancing the function of biopreservation systems by incorporating microbiome studies with innovations, such as nanotechnology. Microbiomes can produce antimicrobial compounds that can be incorporated into nanoparticles to improve food product preservation and controlled–release applications (Hussain et al. 2023). In addition, food waste reduction is achieved using biopreservation to increase the shelf life of food. Microbiome‐dependent preservation methods help decrease losses in the supply chain by maintaining the shelf life of perishable food products. Besides offering fresher consumer products, it helps reduce food waste, as embraced by international practices in a world characterized by a scarcity of resources (Hossain et al. 2024). Microbiomes have practical and functional utility in food ecosystems, including fermentation and biopreservation, and they also possess ecological roles. Microbiota in food matrices refers to relationships among organisms that are beneficial, neutral, and parasitic, and these microorganisms grow favorably under favorable conditions such as pH, temperature, and water activity. Modern and traditional methods of fermentation involve microbiomes to enhance nutrient value, taste, and texture; biopreservation methodology offers safe, natural, and eco‐friendly means for chemical methods of preservation. By applying the potential of microbiomes, the food business can remain a novelty and solve problems related to food safety, quality, and sustainability in a rapidly developing (Table 2) (González et al. 2021; Hernández Medina et al. 2022; Hossain et al. 2024; Hussain et al. 2023).

**TABLE 2:** Microbiome role in food fermentation and biopreservation.

Microbiome role	Description	Examples	References
Starter culture development	Use of selected microbes to initiate and control fermentation	Lactic acid bacteria in yogurt fermentation	(Gregory et al. 2023; Hernández Medina et al. 2022)
Flavor enhancement	Microbial activity contributing to complex flavor profiles in fermented foods	*Saccharomyces cerevisiae* in wine fermentation	(Hossain, Wazed, et al. 2025; Hossain et al. 2024)
Shelf‐life extension	Inhibiting spoilage organisms through fermentation or antimicrobial production	Organic acids from *Lactobacillus* extending kimchi shelf life	(Herrero et al. 2021; Karabulut et al. 2021)
Probiotic development	Enriching foods with beneficial live microbes for health	Probiotic‐rich kefir and kombucha	(Hussain et al. 2023; Infante‐Villamil et al. 2021)
Antimicrobial production	Microbes producing compounds that inhibit pathogens	Bacteriocins from *Lactococcus lactis* against spoilage bacteria	(Jansson et al. 2023; Kern et al. 2021)
Biofilm control	Preventing biofilms on food surfaces using beneficial microbes	*Bacillus subtilis* biofilms reducing fungal spoilage on fruits	(Kabir et al. 2024; Hossain, Didar, et al. 2025)
Nutritional enhancement	Improving food nutrient content through microbial synthesis or bioavailability	Production of vitamin B12 in fermented soy products	(Karpe et al. 2023; Kaul et al. 2017)
Fermentation optimization	Engineering consortia for efficient fermentation processes	Synthetic consortia for ethanol production in biofermentation	(Hossain et al. 2024; Lawson et al. 2019)
Metabolite Production	Generating health‐beneficial metabolites during fermentation	Polyphenols in fermented tea products	(Kovac et al. 2017; McClements et al. 2021)
Biopreservation	Extending food freshness using natural microbial metabolites	Organic acids from *Lactobacillus* preventing spoilage in pickles	(Khoshru et al. 2020; Leggieri et al. 2021)
Stress resistance	Enhancing microbial resilience to food processing conditions	Heat‐tolerant yeast strains in baking	(Ke et al. 2021; Lee et al. 2022)
Enzymatic activity	Producing enzymes to improve food texture or digestibility	Protease production in cheese ripening by *Penicillium* species	(Kaul et al. 2021; Mars et al. 2021)
Carbon source utilization	Microbes converting carbohydrates into fermentation products	Conversion of starch into ethanol by *Zymomonas mobilis*	(Hossain, Wazed, et al. 2025; Mehta et al. 2022)
pH control	Microbial acidification creating inhibitory environments for pathogens	Reduction of pH by *Lactobacillus plantarum* in sauerkraut	(Karabulut et al. 2021; Martínez‐Álvaro et al. 2022)
Alcohol production	Fermentation of sugars into alcohols	*Saccharomyces cerevisiae* in beer production	(Hernández Medina et al. 2022; Mizoguchi 2024)
Aroma formation	Microbial synthesis of aromatic compounds	Production of esters in cider fermentation	(Hossain et al. 2024; Molina et al. 2022)
CO_2_ production	Microbes generating CO_2_ for leavening baked products	*Saccharomyces cerevisiae* in bread dough	(Lawson et al. 2019; Mimee et al. 2016)
Antioxidant production	Enhancing antioxidant levels in fermented foods	Fermented soybean products enriched with antioxidants	(Li et al. 2021; Mizrahi et al. 2021)
Nitrate reduction	Microbial activity reducing nitrates to enhance safety	*Staphylococcus carnosus* in cured meat fermentation	(Kaul et al. 2017; Melara et al. 2022)
Toxin mitigation	Microbes reducing or neutralizing toxins in food	Aflatoxin degradation by specific lactic acid bacteria	(Kabir et al. 2024; Nadarajah and Abdul Rahman 2023)
Protein hydrolysis	Breaking down proteins into bioactive peptides	Casein hydrolysis during cheese fermentation	(Karpe et al. 2023; Naeem and Selamoglu 2024)
Prebiotic utilization	Selective growth on prebiotic substrates	*Bifidobacterium* spp. growing on inulin in functional foods	(Hossain, Wazed, Asha, et al. 2025; Namkung 2020)
Salt tolerance	Adapting to high salinity environments in fermented foods	Halophilic bacteria in soy sauce fermentation	(Jansson et al. 2023; Muszer et al. 2015)
Gas production	Microbes producing gases for food texturization	CO_2_ production in sparkling wine fermentation	(Gregory et al. 2023; Kovac et al. 2017)
Detoxification	Microbial removal of harmful compounds from foods	Cyanide detoxification in cassava fermentation	(Ullah et al. 2025; Mehta et al. 2022)
Food safety	Reducing pathogenic risks through microbial action	*Lactobacillus* spp. lowering *E. coli* populations in fermented sausages	(Mars et al. 2021; Olmo et al. 2022)
Synergistic interactions	Collaborative roles between multiple microbes enhancing fermentation	Co‐fermentation by *Lactobacillus* and yeast in sourdough bread	(Hussain et al. 2023; McKenna et al. 2020)
Pathogen inhibition	Suppressing harmful microbes during food processing	Inhibition of *Salmonella* in fermented dairy products by starter cultures	(Kusstatscher et al. 2019; Mitter et al. 2019)

## 4 Microbiome‐Driven Approaches for Sustainable Food Systems

In this regard, microbiomes play a crucial role in radicalizing new agricultural practices, reducing the impact of environmental effects, and optimizing food production processes. Microbiomes, also known as microbial inhabitants, help in waste conversion and upcycling processes and enhance plant stability and performance, efficient management of nutrients, and overall soil health. More specifically, this section discusses how microbiome engineering might help fix some of the most critical problems of food insecurity and environmental degradation, revitalize eco‐agriculture, utilize waste products, and challenge the dominance of traditional conceptions of protein sources.

### 4.1 Microbiome Engineering for Sustainable Agriculture

The microbiomes that live in the soil and plants enhance crop health and increase agricultural productivity. Phytobiomes are symbiotic plant microbes that improve nutrient acquisition, disease defense, and pollution tolerance. Such microbiomes include the rhizosphere microbiomes, which are those living in the soil surrounding the roots; phyllosphere microbiomes, which are the microbial communities living on different portions of the aboveground plant parts; and endosphere microbiomes, which are those living in the tissues of plants (Olmo et al. 2022). These microbial communities form plant hormones, solubilize phosphorus, and inoculate nitrogen in addition to enhancing plant growth. Therefore, there is a need to minimize the application of synthetic nitrogen fertilizers using nitrogen‐fixing bacteria. For example, Rhizobium species form symbiotic relationships with leguminous plants where atmospheric nitrogen is used to produce nitrogen compounds that are useful for plant growth (Mitter et al. 2019). Microbiome engineering, which involves the optimization and alteration of favorable microbial populations so that plants remain resilient to stress‐like diseases, salinity, and drought, is the primary objective of microbiome engineering (Mehta et al. 2022). This can be achieved using certain microbial strains and mixtures that enhance the health of plants and their ability to deal with stress. Studies have established that some endophytic bacteria and fungi in plants are effective in synthesizing bioactive compounds that enhance pathogen resistance and plant tolerance to abiotic stress (Hossain, Didar, et al. 2025). In addition, improving crop productivity through these microbiome‐driven techniques requires fewer chemical inputs, thereby encouraging sustainability in farming (Table 3).

**TABLE 3:** Microbiome engineering for sustainable agriculture.

Microbiome‐based innovation	Description	Examples	References
Plant growth promotion	Utilizing microbial consortia to enhance nutrient uptake, stress tolerance, and plant productivity	Rhizobacterial inoculants improving nitrogen fixation and nutrient mobilization	(McKenna et al. 2020; Mitter et al. 2019)
Biocontrol agents	Leveraging beneficial microbes to suppress plant pathogens and reduce chemical pesticide use	*Trichoderma* spp. and *Pseudomonas fluorescens* as biological control agents	(Mehta et al. 2022; Olmo et al. 2022)
Soil health improvement	Enhancing soil structure and fertility through microbial‐mediated organic matter decomposition	Use of microbial composting and biofertilizers in degraded soils	(Mehta et al. 2022; Nadarajah and Abdul Rahman 2023)
Crop residue management	Employing microbial consortia for efficient decomposition of crop residues, reducing greenhouse gases	Fungal consortia breaking down lignocellulosic biomass	(Nadarajah and Abdul Rahman 2023; Radaic and Kapila 2021)
Stress resilience engineering	Introducing microbes to mitigate abiotic stress (drought, salinity) effects on crops	Endophytes like Piriformospora indica for improving drought tolerance	(Mehta et al. 2022; Mitter et al. 2019)
Biofertilizers	Use of microbes to mobilize nutrients like phosphorus, potassium, and zinc in soil	Phosphate‐solubilizing bacteria (PSB) for enhanced phosphorus uptake	(Nadarajah and Abdul Rahman 2023; Sidorova and Voronina 2020)
Carbon sequestration	Microbial systems improving carbon capture and storage in agricultural soils	Mycorrhizal fungi aiding carbon retention in soil organic matter	(Mehta et al. 2022; Mitter et al. 2019)
Methane emission reduction	Reducing methane emissions from agricultural activities through microbial manipulation	Methanotrophic bacteria in rice paddies lowering methane release	(Mitter et al. 2019; Mizrahi et al. 2021)
Pest control	Development of microbial‐based tools for integrated pest management	*Bacillus thuringiensis* formulations against insect pests	(Mitter et al. 2019; Qadri et al. 2020)
Waste valorization	Converting agricultural waste into biofertilizers or bioenergy using microbial processes	Anaerobic digestion of crop residues to produce biogas and organic fertilizers	(Mehta et al. 2022; Pereyra‐Camacho and Pardo 2024)
Sustainable fermentation	Innovations using microbial fermentations for bioproducts in agriculture	Lactic acid bacteria‐based plant fermentations for enhanced shelf life	(Molina et al. 2022; Ru et al. 2020)
Microbial bioremediation	Deploying microbes to detoxify soils contaminated by pollutants like heavy metals or pesticides	Bacteria‐assisted remediation of soils with high arsenic or cadmium content	(Mehta et al. 2022; Sessitsch et al. 2023)
Precision agriculture tools	Integration of microbial innovations with digital tools for tailored agricultural solutions	Machine learning models optimizing microbial consortia for specific crops	(Nadarajah and Abdul Rahman 2023; Topçuoğlu et al. 2020)
Plant microbiome editing	Engineering microbiomes to enhance beneficial interactions while suppressing harmful microbes	CRISPR‐based microbiome manipulation to boost rhizosphere efficiency	(Mehta et al. 2022; Thakur et al. 2023)
Nutritional fortification	Leveraging microbial systems to enhance the nutritional profile of crops	Microbiota‐assisted biofortification of crops with iron or zinc	(Mehta et al. 2022; Sidorova and Voronina 2020)
Biodegradable pesticides	Development of environmentally friendly microbial pesticides to reduce chemical use	Beauveria bassiana formulations as biodegradable insecticides	(Mehta et al. 2022; Olmo et al. 2022)
Livestock microbiome engineering	Modulating livestock gut microbiomes to improve feed efficiency and reduce environmental impact	Probiotic supplements for cattle to reduce methane emissions	(McKenna et al. 2020; Mizrahi et al. 2021)
Microbial inoculants for aquaponics	Use of beneficial microbes to optimize nutrient cycling in aquaponics systems	Nitrosomonas bacteria enhancing nitrogen cycling in aquaponics	(Mehta et al. 2022; Rimoldi et al. 2018)
Biofilm‐based delivery systems	Creating biofilm‐based microbial systems for efficient nutrient or pesticide delivery	Biofilm carriers delivering beneficial rhizobacteria to crops	(Mitter et al. 2019; Stoddard et al. 2024)
Systemic resistance induction	Harnessing microbes to activate systemic resistance against diseases in crops	Induction of systemic resistance in tomato plants by *Bacillus subtilis*	(Nadarajah and Abdul Rahman 2023; Singh and Trivedi 2017)

### 4.2 Biofertilizers and Biopesticides: Reducing Chemical Input in Food Production

Their harm to the environment, as well as their impact on human health, calls for the utilization of biofertilizers and biopesticides from plant and soil microbes (Melara et al. 2022). The number of nutrients available for plant growth is increased by the microorganisms present in the biofertilizers. Some examples are phosphorus‐solubilizing bacteria, which, when applied naturally without chemical fertilizers, can convert insoluble phosphorus into forms that plants can take, thus enhancing the fertility of the soils and overall crop yields (Mehta et al. 2022). However, biopesticides use microbial antagonists that interfere with plant diseases or pests in many ways, such as competition for resources, production of antimicrobial compounds, or stimulation of plant defense mechanisms (Lawson et al. 2019). Another approach is the application of Bacillus species, which synthesize lipopeptides to suppress fungal growth (Hossain, Didar, et al. 2025). Additionally, mutualistic associations between plants and other microorganisms, such as mycorrhizal fungi, enhance nutrient and water acquisition for the plant, while simultaneously enhancing a protective coating to the roots against disease‐causing pathogens (Nadarajah and Abdul Rahman 2023).

Biofertilizers and biopesticides derived from an understanding of the crop microbiome work toward decreasing the agricultural environmental impacts that support soil health and toward helping build more sustainable crop production by minimizing the use of chemical fertilizer and pesticide inputs. These inputs translate into the sustainability of diverse, environmentally friendly global endeavors in conserving diversities, boosting food chain security, and reducing chemical hazards (Mitter et al. 2019; Naeem and Selamoglu 2024). In addition to biofertilizers and biopesticides, traditional soil management practices such as composting, intercropping, and organic amendments also play an important role in shaping plant microbiomes. Such traditional management practices have been widely adopted by farmers in Africa, Asia, and Latin America and remain fundamental for building resilient agricultural systems in the absence of high‐tech microbial options (Hanif et al. 2024).

### 4.3 Waste Valorization and Upcycling With Microbial Consortia

Food waste leads to resource waste and contributes to the emission of greenhouse gases, making it a major environmental issue. Microbial forms that consist of different microbial species in symbiotic relationships offer a unique approach to repurposing food waste products as fertilizers, biofuels, and bioplastics (Paul et al. 2023). The process of converting food waste into simple molecules via a microbiome capable of processing it into value‐added products is called microbiome‐based waste valorization. For instance, waste foods are converted into biogas, a renewable energy source, using microbial societies in anaerobic digestion processes (Lawson et al. 2019). In addition, some bacteria can metabolize waste into alcohols and other organic acids, which are used to manufacture biopolymers. The negative environmental impacts of waste food are reduced by this technique, in addition to creating a circular bioeconomy of turning trash into valuable products (Hossain, Wazed, Asha, et al. 2025). In addition, the same microbial consortium is being explored for its ability to convert agricultural waste into biofertilizers and soil conditioners for practicing sustainable agriculture. Municipal food waste streams may be used in microbial fermentation processes to reduce the effects of the food sector on the environment while creating essential products for business and agriculture (Table 4) (Pereyra‐Camacho and Pardo 2024).

**TABLE 4:** Waste valorization and upcycling with microbial consortia.

Application	Description	Examples	References
Plastic waste degradation	Microbes degrading synthetic plastics into simpler compounds for reuse	*Pseudomonas* spp. breaking down polyethylene	(Ru et al. 2020; Schaerer et al. 2024)
Food waste upcycling	Microbial fermentation to convert food waste into value‐added products	Bioconversion of food scraps into animal feed	(Wang, Wang, Xiao, et al. 2024; Wassermann et al. 2022)
Agricultural waste biorefining	Utilizing microbes to produce biofuels and bioplastics from agricultural residues	Ethanol production from corn stover using fungal consortia	(Ru et al. 2020; Stoddard et al. 2024)
Biochar production enhancement	Microbial treatments improving biochar properties for soil enrichment	Biochar functionalized with phosphate‐solubilizing bacteria	(Rändler‐Kleine et al. 2020; Srivastava et al. 2021)
Organic waste composting	Accelerating composting with microbial inoculants	Rapid decomposition of kitchen waste by Bacillus strains	(Rimoldi et al. 2018; Saleem and Saleem 2015)
Lignocellulose breakdown	Microbes degrading lignocellulosic biomass into fermentable sugars	Cellulase‐producing fungi for converting rice husks into sugars	(Rändler‐Kleine et al. 2020; Satta et al. 2024)
Bioplastic synthesis	Microbial production of biodegradable plastics from waste streams	Polyhydroxyalkanoates (PHA) synthesis from food industry waste	(Ru et al. 2020; Sessitsch et al. 2023)
Textile waste valorization	Converting discarded textiles into bioproducts using microbial consortia	Biocatalytic conversion of polyester waste into monomers	(Ru et al. 2020; Stoddard et al. 2024)
E‐waste bioremediation	Microbial extraction of metals from electronic waste	Recovery of gold using cyanogenic bacteria	(Saleem and Saleem 2015; Srivastava et al. 2021)
Greenhouse gas mitigation	Microbial systems reducing emissions from organic waste	Methanotrophs in landfill biocovers lowering methane release	(Sariola and Gilbert 2020; Sidorova and Voronina 2020)
Protein‐rich feed production	Upcycling organic waste into high‐protein animal feed using microbes	Black soldier fly larvae farming facilitated by microbial fermentation of food waste	(Wang, Wang, Xiao, et al. 2024; Wassermann et al. 2022)
Nutrient recovery	Microbial recovery of phosphorus and nitrogen from waste streams	Anaerobic digestion of wastewater for nutrient‐rich effluents	(Ru et al. 2020; Saleem and Saleem 2015)
Bioethanol production	Fermentation of agricultural by‐products into bioethanol by microbial consortia	Yeast‐based fermentation of sugarcane bagasse	(Srivastava et al. 2021; Wassermann et al. 2022)
Paper waste recycling	Microbial enzymes breaking down paper waste for reuse	Xylanase‐producing bacteria facilitating paper pulp recycling	(Rändler‐Kleine et al. 2020; Schaerer et al. 2024)
Industrial wastewater treatment	Decontaminating industrial effluents using microbial biofilms	Removal of heavy metals by sulfate‐reducing bacteria	(Sessitsch et al. 2023; Trif et al. 2022)
Microalgae for CO_2_ fixation	Using algae to upcycle CO_2_ into biofuels or biomass	*Chlorella* spp. converting CO_2_ emissions into biodiesel	(Ru et al. 2020; Wang, Wang, Liu, et al. 2024)
Landfill waste management	Deploying microbial consortia for landfill waste decomposition	Consortia improving methane oxidation in landfill sites	(Satta et al. 2024; Stoddard et al. 2024)
Waste‐derived biosurfactants	Producing biosurfactants from waste substrates for industrial applications	Rhamnolipids from cooking oil waste using *Pseudomonas aeruginosa*	(Ru et al. 2020; Singh et al. 2018)
Plastic upcycling	Engineering microbes to upcycle plastic waste into high‐value chemicals	Engineered Ideonella sakaiensis converting PET into terephthalic acid	(Ru et al. 2020; Stoddard et al. 2024)
Urban waste valorization	Utilizing microbial technologies for processing municipal solid waste	Fermentation of urban organic waste into biohydrogen	(Saleem and Saleem 2015; Srivastava et al. 2021)

### 4.4 Innovations in Upcycling: Production of High‐Value Compounds

Alcohol fermentation using microbial consortia for the effective management and upcycling of food waste opens up opportunities for the synthesis of valuable chemicals, such as bioactive peptides, enzymes, and other nutraceuticals. These substances have applications in pharmaceutical, food, and cosmetic industries. It has also been ascertained that the efficiency of biomass conversion can be enhanced by engineering microbial consortia in microbial fermentation processes to generate enzymes that break down plant components (Paul et al. 2023). In recent years, significant developments in microbiome engineering and synthetic biology have been made to develop microbial biosynthetic platforms for the production of health‐promoting bioactive peptides. These peptides have been shown to reduce inflammation, improve gut health, and prevent chronic diseases (Lawson et al. 2019). In addition, genetically modified microbes are helpful for manufacturing novel antimicrobial agents, and food preservatives are capable of synthesizing antimicrobial peptides (Paul et al. 2023). Trash upcycling not only results in increased repayment of food waste to obtain a higher market value reflected in the value of these chemicals, but also promotes the application of sustainable biotechnologies associated with circular economy concepts. This innovative method of waste valorization shows that microbiome‐driven processes are crucial for addressing issues such as food waste, resource depletion, and climate change (Hossain, Wazed, Asha, et al. 2025).

### 4.5 Microbiomes in Alternative Protein Production

Emerging concerns about the impacts of conventional animal farming practices on the environment continue to fuel the growth of the global market for pork. Novel proteins generated through fermentation comprise mycoproteins, microbial proteins, and single‐cell proteins, and are considered viable for fulfilling protein requirements in the future (Mizrahi et al. 2021). These protein sources are derived from microorganisms that can be cultivated from food waste substrates and renewable feedstocks such as fungi, bacteria, and algae. This means that microbial biomass is subjected to fermentation to produce microbial proteins, which can be adopted as proteins derived from the microbial biome and incorporated into protein foods intended for human consumption. Mycoproteins are produced by filamentous fungi and therefore have a higher protein content than fats. They are more environmentally friendly than conventional meat production methods. They are already employed in commercially available meat substitutes (Lawson et al. 2019). Another promising method of producing proteins for humans and animals with scarce land and water is the production of single‐cell proteins from algae and bacteria (Afridi et al. 2022). Therefore, fermentation‐based protein production can be regarded as a scalable and sustainable means to address the growing world population's protein demands. In addition, these alternative proteins can be manufactured from renewable feedstock, such as food waste or agricultural residues, and thus, should support waste reduction and circular economy strategies (Table 5) (Hossain, Wazed, Asha, et al. 2025; McClements et al. 2021).

**TABLE 5:** Microbiome in alternative protein production.

Protein source	Description	Examples	References
Microalgae	Microalgae are a promising protein source, enriched with essential amino acids. Microbiome interactions enhance yield and sustainability	Spirulina, Chlorella	(Ballerstedt et al. 2021)
Insects	Insect farming for protein is gaining attention, with microbiomes influencing growth and health	Crickets, Mealworms	(Bao et al. 2023)
Fungi	Fungal‐based proteins, such as mycoprotein, utilize microbiomes for enhanced fermentation processes	Fusarium venenatum	(Beattie et al. 2024)
Bacteria	Bacteria can be engineered to produce proteins via fermentation. The microbiome aids in process optimization	*Bacillus subtilis* , *E. coli*	(Beck et al. 2021)
Yeast	Yeast‐based protein production uses microbiome interactions for fermentation efficiency	*Saccharomyces cerevisiae*	(Ferone et al. 2020)
Algae	Algae are used for high‐protein biomass production, with microbiomes influencing growth	*Haematococcus pluvialis*	(Berg et al. 2020)
Plant‐based proteins	Microbiomes support plant protein production by enhancing soil quality and plant health	Soy, Peas, Lentils	(Hossain, Wazed, Shuvo, et al. 2025)
Seaweed	Seaweed‐based proteins benefit from microbiomes in nutrient cycling and growth	Kelp, Wakame	(Carmody and Bisanz 2023)
animal cells	Cultured meat production is influenced by microbiomes during cell growth and differentiation	Cultured Beef	(Catania et al. 2021)
Spore‐based proteins	Microbiome‐driven fermentation processes help in the production of spore‐based proteins	Rhizopus, Aspergillus	(Compant et al. 2024)
Precision fermentation	Microbiomes in precision fermentation systems optimize protein synthesis from microbial cells	Recombinant Proteins	(Siddiqui et al. 2025)
Mycoprotein	Fungi‐based protein using microbiomes for enhanced yield and efficiency in fermentation	Quorn	(De Filippis et al. 2021)
Microbial consortia	Combining multiple microbial species enhances protein production and nutrient cycling	Mixed Cultures	(De Vrieze et al. 2018)
Synthetic biology	Engineering microbiomes for efficient protein production using genetic modifications	Genetically Engineered Microbes	(Dey and Ray Chaudhuri 2023)
Metabolic engineering	Microbiomes in metabolic engineering aid in the optimization of protein biosynthesis pathways	Modified Bacteria	(Dubey and Kumar 2022)
Probiotics	Probiotic microbes are used in alternative protein production, influencing gut microbiomes	Lactobacillus, Bifidobacterium	(Eleftheriadou et al. 2017)
Bioreactor systems	Microbial interactions in bioreactor systems optimize alternative protein production	Bioreactor‐based Fermentation	(El‐Metwally et al. 2023)
Hydroponics	Hydroponic systems with microbiomes promote plant protein growth	Hydroponic Soybeans	(French et al. 2021)
In vitro meat	The microbiome in cell culture media can influence protein production in cultured meat	Lab‐grown Meat	(Frey‐Klett et al. 2011)
Protein hydrolysates	Microbiomes involved in the fermentation of proteins into hydrolysates can enhance protein quality	Protein Hydrolysates from Soy	(García‐Depraect et al. 2021)
Fermentation products	Microbiome‐driven fermentation processes produce protein‐rich products	Tempeh, Miso	(Ghannam and Techtmann 2021)

### 4.6 Enhancing Nutritional Profiles and Reducing Off‐Flavors Through Microbiome Manipulation

Although fermentation‐based alternative proteins have many positive impacts on the environment and human health, they have problems with flavor, texture, and nutritional value. Manipulation of the microbiome has proven to be a functional tactic for improving the nutritional value and sensory appeal of such substitute proteins. Such flavor profiles of microbial proteins can be enhanced by engineering microbial consortia or by selecting specific strains that possess acceptable metabolic capabilities, thereby reducing the off‐flavor issues associated with fermentation‐based products (Hossain, Didar, et al. 2025; Lee et al. 2022). In addition to improving flavor, microbiome engineering can also improve the nutritional profile of alternative proteins by enhancing the bioavailability of critical amino acids, vitamins, and minerals. Indeed, alternative protein sources can have equivalent nutritional profiles to animal‐derived proteins because, for example, some microbial species can be engineered for the biomanufacturing of vitamin B12. This component is often absent in plant‐based diets (Lawson et al. 2019). More importantly, the design of microbial consortia can do much to optimize protein content and minimize anti‐nutritional factors to produce an improved quality and digestibility alternative protein product (Nadarajah and Abdul Rahman 2023). The food industry could eliminate severe problems in global food security and sustainability through the development of microbiome‐driven alternative proteins that are not only more sustainable but also nutritionally superior and more appealing to consumers (McClements et al. 2021; Mizrahi et al. 2021). The microbiome‐driven approach offers new solutions to problems in agriculture, food waste management, and alternative protein production; hence, it challenges established sustainable food systems. There is excellent potential for microbiomes to improve resilience among crops, develop sustainable protein sources, and minimize environmental degradation due to food production. Waste valorization, fermentation‐based alternative proteins, and microbiome engineering are combinations in which microbial consortia might drive a more viable and robust global food system. Synthetic biology and microbiome research are continuously improving, and microbial communities have made a more outstanding contribution to make toward pressing issues at stake involving environmental conservation, nutrition, and food security to ensure the sustainability of food in the future (Hossain, Didar, et al. 2025; Hossain, Wazed, Asha, et al. 2025; McClements et al. 2021; Mitter et al. 2019; Olmo et al. 2022).

## 5 Integrating Multi‐Omics and Systems Biology in Microbiome Research

Systems biology and the integration of multi‐omics approaches have marked a revolution in microbiome studies, underpinning the immense complexity of microbial communities with their dynamic relationships to the environment. The integration of techniques, including machine learning, metatranscriptomics, and metaproteomics, will allow better capture of dynamic microbial responses and can offer predictions of their behaviors. Ultimately, these benefits will result in improved output in agriculture, food systems, and personalized health interventions. These novel approaches provide the foundation necessary for the development of durable and effective strategies to optimize the performance of microbiomes in different environments (Table 6).

**TABLE 6:** Multi‐omics approaches in microbiome research.

Omics technology	Description	Examples	References
Genomics	Study of the genetic material (DNA) of microorganisms in the microbiome	Sequencing of microbial genomes to identify species composition	(Gregory et al. 2023)
Metagenomics	Analysis of the genetic material recovered directly from environmental samples	Identifying microbial communities in soil or gut	(Hossain, Wazed, et al. 2025)
Transcriptomics	Study of RNA expression to analyze microbial activity	Gene expression in response to environmental stressors	(Hernández Medina et al. 2022)
Proteomics	Study of proteins expressed by microbiomes to understand functionality	Profiling of microbial proteins in the human gut microbiome	(Herrero et al. 2021)
Metabolomics	Identification and quantification of metabolites from microbiome samples	Identifying metabolites involved in fermentation processes in the gut	(Hossain et al. 2024)
Lipidomics	Study of lipid profiles in microbial communities	Lipid profile changes in human gut microbiota under different diets	(Hussain et al. 2023)
Glycomics	Analyzing the sugars and glycans produced by microbiomes	Glycan profiles in microbiome‐host interactions	(Infante‐Villamil et al. 2021)
Metaproteomics	Characterizing the protein functions of a microbiome's community	Profiling the protein changes during plant‐microbe interactions	(Jansson et al. 2023)
Epigenomics	Analysis of microbial epigenetic modifications	Microbial DNA methylation in response to stress	(Kabir et al. 2024)
Exposomics	Study of environmental exposures and their influence on the microbiome	Effects of pesticide exposure on soil microbiomes	(Karabulut et al. 2021)
Metatranscriptomics	Combining transcriptomics with metagenomics to study microbiome functions	Assessing the functional potential of microbial communities in the gut	(Kaul et al. 2021)
Phenomics	Study of physical and biochemical traits influenced by the microbiome	Phenotypic changes in crops due to microbial inoculation	(Ke et al. 2021)
Single‐cell omics	Analyzing the gene expression and functions of individual cells in microbiomes	Studying rare microbial species in complex environments	(Kern et al. 2021)
Imaging metabolomics	Use of imaging techniques to map metabolites in microbiomes	Imaging the distribution of metabolites in the gut microbiome	(Khoshru et al. 2020)
Environmental metagenomics	Use of metagenomics to study the impact of environmental factors on microbiomes	Exploring microbial communities in polluted environments	(Kovac et al. 2017)
Structural genomics	Analysis of microbial protein structures to understand functionality	Determining protein structures to study microbial metabolic pathways	(Hossain, Didar, et al. 2025)
Functional genomics	Study of gene functions and their impact on microbiome activity	Investigating the roles of specific genes in microbial interactions	(Kusstatscher et al. 2019)
Comparative genomics	Comparing microbial genomes to identify differences in traits	Comparison of microbial communities in healthy vs. diseased plants	(Lawson et al. 2019)
Systems biology	Using a systems approach to understand microbiome interactions	Modeling microbiome functions in human health	(Lee et al. 2022)
Bioinformatics	Application of computational tools to interpret omics data	Using bioinformatics tools to analyze microbiome sequencing data	(Hossain, Wazed, Asha, et al. 2025)
Data integration	Combining various omics data for a holistic microbiome analysis	Integrating genomics, proteomics, and metabolomics to understand microbiomes	(Leggieri et al. 2021)

### 5.1 Metatranscriptomics and Metaproteomics

Metaproteomics and metatranscriptomics are two methods applied to identify the metabolic activity currently occurring within microbial communities. While metagenomics may provide insights into the genetic potential of microbiomes, real‐time information on protein synthesis and gene expression comes from metatranscriptomics and metaproteomics. These techniques are more enlightening in learning how different environments, such as soils, the human gut, or unique food production settings, influence the composition and functional traits of microbiomes. These methods have enabled researchers to explore how microbiomes adapt to various dietary backgrounds by monitoring temporal variations in microbial gene expression or protein production. For example, metatranscriptomic studies of agricultural microbiomes can reveal how nutrient inputs, biofertilizers, and plant exudates change the expression of soil bacteria and fungi, enabling improved nutrient cycling and plant development.

Pinu et al. 2019 indicated that metaproteomics provides information on shifting microbial communities at the protein level. This is a vital component in determining the functions of microbes in nutrient mobilization and degradation processes (Pinu et al. 2019). The applications of these findings range from better microbial treatments in food production systems to stimulating soil health under sustainable agricultural methodologies (Pinu et al. 2019). Most importantly, microbial community studies in animal stomachs have already identified the importance of metaproteomics in uncovering essential proteins participating in nutrition absorption and digestion. Using next‐generation sequencing technologies, Rimoldi et al. (2018) explored the gut microbiome of the rainbow trout. They described that its metaproteome changed with different protein sources and animal byproducts fed to fish (Rimoldi et al. 2018). This article provides knowledge that will underpin the development of sustainable aquaculture methods through the optimization of feed formulation, enabling animal health and environmental sustainability. Knowledge acquired through metatranscriptomic and metaproteomic findings can be translated into industrially useful information by designing microbial consortia for bioremediation and biodegradation purposes. For instance, these approaches have been applied in the study of microbial populations involved in the degradation of plastic waste with the aim of identifying the exact enzymes that transform plastic polymers into more eco‐friendly substances (Ru et al. 2020; Satta et al. 2024). Starting from wastes for the production of valuable byproducts using microbes, this type of research offers practical solutions to solve environmental problems associated with plastic pollution. Hence, to precisely intervene in and enhance the performance of microbial communities in agriculture, food systems, and environmental management, metatranscriptomics and metaproteomics must be performed to capture the dynamic activities of microbiomes (Qadri et al. 2020; Rändler‐Kleine et al. 2020; Salaheen et al. 2017).

### 5.2 Machine Learning and Predictive Modeling in Microbiome Research

Rapid developments in microbiome studies have greatly improved the capability to understand complex microbial datasets through machine learning and predictive modeling, thus allowing for more accurate prediction of the behavior of microbiomes and their interactions. These methods are needed for applications such as customized nutrition, agriculture, and environmental sustainability because they provide a profound understanding of the multifaceted relationships between microbes, their hosts, and their environments (Saleem and Saleem 2015). Large‐scale datasets from multi‐omics research, including metabolomics, metagenomics, and metatranscriptomics, are employed by machine‐learning algorithms to identify patterns and create prediction models. This has a more significant effect on agriculture in developing machine learning models to forecast how microbiomes react to other inputs, such as fertilizers and biopesticides. For instance, machine learning models have been developed to predict how plant microbiomes affect crop yields to inform farmers of the best microbial inoculants they might use in a quest to improve productivity with minimal chemical use (Saleem and Saleem 2015) (Singh and Trivedi 2017). Machine learning has become an integral approach in personalized nutrition to investigate the interplay between microbiome composition, diet, and health outcomes. Microbiome data enable predictive models to assess how certain foods or nutrients contribute to a person's microbiome and, conversely, how those microbiome changes affect health and are integrated with dietary information. By applying the approach, personalized diet recommendations with improved metabolic functions, gut health, and even prevention from diabetes and obesity could be yielded (Salaheen et al. 2017; Thakur et al. 2023). Topçuoğlu et al. (2020) proposed a framework for applying machine learning to microbiome classification challenges, which addresses the problem of the high dimensionality of microbiome datasets. Their research has shown that ML methods using random forests and SVMs can enhance diagnosis and therapy based on microbiomes through the correct classification of microbial populations (Topçuoğlu et al. 2020). Such techniques may enable the targeted design of microbiome therapeutics, not only in clinical settings but also in agriculture. Predictive modeling also appeared to be one useful technique that could be used to make sense of microbiomes in environmental applications. Researchers have applied machine learning to simulate microbial interactions that occur during biodegradation and bioremediation. For instance, Schaerer 2023 discussed the potential of enriched microbial consortia for plastic waste upcycling using machine learning algorithms that predict microbial communities with the best ability to degrade certain polymer types. Information is of utmost importance in optimizing the use of biotechnology in applications planned to reduce environmental pollution and create valuable byproducts from waste (Schaerer et al. 2024) (Stoddard et al. 2024). In agriculture, machine learning models find applications in forecasting plant‐microbe interactions to predict the outcome of crop health and stress resistance. Microbiome‐based predictive models can contribute toward food and nutrient security by enhancing disease resistance and nutrient deficiency in crops (Singh and Trivedi 2017). Furthermore, to identify microbial consortia that can promote plant growth under less favorable conditions, such as salinity or drought, these models have the potential to contribute to the establishment of more eco‐friendly approaches (Trif et al. 2022). Machine learning in microbiome research has also been used to study symbiotic relationships between plants and the bacteria with which they are associated (Tsiknia et al. 2021). For example, Tsiknia et al. (2021) investigated how machine learning models assessing multispecies interactions within legume roots promote nitrogen fixation and further soil fertility. This has tremendous implications for sustainable agriculture regarding the reduction of synthetic fertilizers (Sessitsch et al. 2023; Sidorova and Voronina 2020; Singh et al. 2018). In general, the embedding of machine learning with predictive modeling in microbiome research provides new insights into microbial behavior and interactions that allow for better and more successful interventions in several fields, from personalized nutrition to sustainable agriculture and environmental management (Trivedi et al. 2021). Machine learning coupled with predictive modeling and multi‐omics techniques, including metatranscriptomics and metaproteomics, are new frontiers that have revolutionized microbiome research. These advanced methods provide a more profound understanding of microbial function, community dynamics, and interactions with the environment, which will enable novel applications in food systems, agriculture, and personalized health interventions. These advanced technologies have the potential to assist researchers in developing more sustainable, effective, and targeted microbiome‐based solutions for global issues affecting food security, environmental sustainability, and human health (Table 7) (Pinu et al. 2019; Radaic and Kapila 2021; Sariola and Gilbert 2020; Srivastava et al. 2021; Von Braun et al. 2021).

**TABLE 7:** Machine learning in microbiome research.

Application	Description	Examples	References
Disease prediction	Using machine learning models to predict disease outcomes based on microbiome data	Predicting gut‐related diseases from microbiome sequencing data	(Hernández Medina et al. 2022)
Biomarker identification	Identifying microbiome‐based biomarkers for various diseases	Identifying biomarkers for inflammatory bowel disease (IBD)	(Marcos‐Zambrano et al. 2021)
Microbiome profiling	Classifying microbiomes and understanding diversity using machine learning	Classifying microbiomes in healthy vs. diseased individuals	(Hernández Medina et al. 2022)
Predictive modeling	Developing predictive models to understand microbiome behavior and interactions	Predicting gut microbiome responses to diet changes	(Lawson et al. 2019)
Host‐microbiome interaction	Using machine learning to study interactions between host and microbiome	Studying gut‐brain axis and microbiome relationships	(Karabulut et al. 2021)
Antibiotic resistance prediction	Predicting and identifying genes associated with antibiotic resistance	Modeling antibiotic resistance based on microbiome composition	(Hernández Medina et al. 2022)
Microbiome‐ecology modeling	Applying machine learning to model ecological processes within microbiomes	Modeling ecosystem dynamics in soil microbiomes	(Lee et al. 2022)
Microbial interaction networks	Inferring microbial interaction networks to understand microbiome functions	Building co‐occurrence networks for gut microbiota	(Lee et al. 2022)
Strain‐level classification	Identifying and classifying microbiome strains at the genomic level	Strain‐level classification of gut microbiome diversity	(Hernández Medina et al. 2022)
Drug response prediction	Predicting individual drug responses based on microbiome composition	Predicting cancer treatment responses based on the microbiome	(Leggieri et al. 2021)
Precision medicine	Integrating microbiome data into personalized treatment strategies	Personalized nutrition recommendations based on microbiome analysis	(Mars et al. 2021)
Metabolic pathway prediction	Predicting microbial metabolism and functional pathways in the microbiome	Predicting gut microbial metabolism of dietary fibers	(Leggieri et al. 2021)
Microbiome‐based diagnostics	Using microbiome data for diagnostic purposes	Using microbiome signatures to diagnose infectious diseases	(Marcos‐Zambrano et al. 2021)
Epidemiological forecasting	Forecasting disease outbreaks and microbiome changes using machine learning	Forecasting epidemics based on environmental and microbiome factors	(Leggieri et al. 2021)
Treatment response modeling	Modeling how different microbiomes respond to medical treatments	Predicting microbiome changes after chemotherapy treatment	(Leggieri et al. 2021)
Ecological and environmental modeling	Using machine learning to study environmental impacts on microbiomes	Modeling effects of climate change on soil microbiome composition	(Jansson et al. 2023)

## 6 Microbiome Innovations for Food Safety and Quality Enhancement

Food safety and its enhancement in quality have become of increasing importance, with motivation being inspired by a quest to seek more dependable, sustainable, and effective means of detecting and managing foodborne pathogens and in so far as the application of microbiome advances is concerned. Such advances include microbial biosensors for disease detection in real‐time applications using natural and modified microorganisms as well as biocontrol techniques. These methods are foreseen to help both the food business and consumers maintain and extend food quality and enhance food safety management systems (Berg et al. 2020; Hossain, Wazed, Shuvo, et al. 2025).

### 6.1 Microbial Biosensors for Real‐Time Pathogen Detection

Microbial biosensors are expected to contribute to real‐time pathogen detection by providing fast, trustworthy, and inexpensive means of ensuring food safety. Microbial biosensors take advantage of the capacity of microorganisms to yield quantifiable signals after exposure to specific stimuli to detect adulterants, toxins, or foodborne pathogens. Food industries can confidently monitor food safety consistently through microbial biosensors, which allows for identification and prevention at an early stage to avoid contamination events that might affect public health (Carmody and Bisanz 2023; Catania et al. 2021). The development of microbial biosensors involves a combination of enzymes or whole cells with physical transducers to detect and measure food pathogens. These biosensors are extremely sensitive and can be developed for a wide range of bacteria that are often responsible for foodborne diseases, such as Salmonella, *Listeria monocytogenes* , and *Escherichia coli* (Berg et al. 2020). Biosensors have the potential to be embedded within management systems for food safety along the entire value chain of food production, from production and packaging to actual distribution, with the aim of continuously monitoring and updating data to help maintain control over contamination levels (Compant et al. 2024). Another relevant case study in the application of food safety biosensors is the use of microbial biosensors to check for the presence of Listeria species in dairy processing plants. These biosensors, through the enablement of real‐time identification of the pathogen in milk samples, reduce the possibility of contamination and improve overall product safety (Siddiqui et al. 2025). Such microbial biosensors have also been applied to detect contamination by Salmonella in meat processing plants to ensure the safety of meat products and regulatory compliance (De Filippis et al. 2021). The applications of microbial biosensors are not limited to the identification of pathogens; they can also be used to determine the freshness and quality of food. For instance, freshness in seafood and other perishable products can be detected using biosensors that detect spoilage compounds, such as hydrogen sulfide or ammonia. Besides promoting food safety, this contact will provide timely information about spoilage, thereby enabling increased product shelf life and improved food inventory management (De Vrieze et al. 2018).

However, microbial biosensors have drawbacks. For example, they require further miniaturization in terms of size and cost for better applicability in the food industry. Nevertheless, it is a promising tool for food safety and quality control in the future, as continuous research and technical development have increased sensitivity, specificity, and usability (Dey and Ray Chaudhuri 2023; Dubey and Kumar 2022).

### 6.2 Biocontrol Strategies Using Native and Engineered Microbes

Other developments in biocontrol techniques include the use of native and artificial bacteria to enhance food safety and quality. This will allow the prevention of foodborne pathogens or spoilage organisms by employing desirable microorganisms for increased food product safety and longer shelf life. Native microbiomes are naturally present in food environments, together with modulating microorganisms engineered for pre‐defined biocontrol activities, non‐toxic alternatives to conventional chemical preservatives, and pesticides (Eleftheriadou et al. 2017; El‐Metwally et al. 2023). Native microbiomes consist of numerous microbial communities that exist in the environment. They greatly contribute to maintaining the ecological balance and, at the same time, serve to terminate the penetration area of some dangerous infections. Such naturally occurring microbes can be utilized to minimize the risk in food production systems by outcompeting noxious germs that contaminate and spoil. Fermented foods such as sauerkraut and yogurt can be uncontaminated with naturally occurring lactic acid bacteria, which can prevent the growth of unwanted bacteria, such as *Listeria monocytogenes* and *Escherichia coli* (French et al. 2021). In addition, research related to utilizing natural microbiomes as biocontrol agents in the postharvest management of fruits and vegetables has also been conducted. For instance, several studies have shown that naturally occurring yeasts on citrus have been inhibited in the development of fungal infections, such as those from *Penicillium* spp., which are responsible for significant postharvest losses (Frey‐Klett et al. 2011). Similarly, leafy greens have also been protected against contamination by pathogenic bacteria, such as *Salmonella* and *E. coli* , using wild‐type bacterial strains isolated from the phyllosphere or the microbial population residing on the leaf surface (García‐Depraect et al. 2021). Another significant advantage of using native microbiomes for biocontrol is that their potential to adapt to specific environmental conditions enables them to effectively control pathogens in various food production systems. The application methods for these microbiomes could be quite flexible and could be integrated into packaging materials, sprayed, or coated (Ghannam and Techtmann 2021).

### 6.3 Engineered Microbial Solutions for Enhancing Food Shelf Life and Safety

In the recent era, engineered microorganisms, apart from naturally occurring microbiomes, have been considered for providing proficient levels of improvement in food safety and quality. Through genetic engineering and synthetic biology, it is now easy to develop microbial strains that can be specifically engineered to target foodborne pathogens or enhance food preservation. Through genetic design, such modified microorganisms can produce antibacterial compounds, detoxify toxins, and improve the nutritional value of food products (Ghannoum et al. 2021; González et al. 2021). Engineered microbial approaches have also utilized genetically engineered probiotic bacteria to express bacteriocins, which are antimicrobial peptides that inhibit the growth of foodborne pathogens. These bacteriocin‐producing probiotics have been incorporated into various dairy foods, such as yogurt and cheese, providing extended shelf life and reduced risk of contamination by harmful bacteria, such as Salmonella and Listeria (Gopal and Gupta 2019). Another application of modified bacteria is the production of non‐spoiled foods. For example, the utilization of ethanol and organic acid‐producing transgenic yeasts has extended the shelf life of various bakery products and beverages by inhibiting the growth of spoiled organisms (Graw et al. 2021). The application of biofilm‐degrading enzymes from engineered bacterial strains has been considered in the context of meat preservation as an approach to prevent contamination and spoilage. Biofilms are varnish‐like layers produced on the surface by microbial communities. They pose a grave menace to the food processing area because they act as havens for injurious bacteria. Such microbes clean and keep the equipment used in the processing of food free from bacteria by degrading biofilms (Gregory et al. 2023). Food safety might also be enhanced by the use of genetically engineered microorganisms against novel pollutants, such as chemical residues and antibiotic‐resistant bacteria. Researchers have attempted to reduce the incidence of foodborne illnesses and make food products generally safer by developing bacteria that can degrade or inactivate these pollutants (Hossain, Wazed, et al. 2025; Hernández Medina et al. 2022). Although applications of improved microorganisms for food safety are still in their infancy, new research and regulations allow for more potential uses. Given the growing demand for natural and ecological products, there is an urgent need in the food industry for these bacteria to work effectively and efficiently to replace traditional chemical preservatives and antimicrobials (Herrero et al. 2021). The latest developments in the domain of microbiomes include microbial biosensors that are capable of real‐time disease detection and biocontrol techniques that utilize native and artificially modulating bacteria for food safety and quality enhancement. These methods provide feasible, economical, and long‐term approaches to prevent foodborne diseases, extend the shelf life, and maintain food quality. Challenges associated with the provision of safe and high‐quality food to the increasing global population will be addressed by integrating these tools into food safety management systems, as research and technical improvements continue to fine‐tune them (Berg et al. 2020; Hossain, Wazed, Shuvo, et al. 2025; Catania et al. 2021; Compant et al. 2024; Ghannam and Techtmann 2021; Ghannoum et al. 2021).

## 7 The Intersection of Microbiome Science and Consumer Health

### 7.1 Microbiome‐Based Functional Foods and Supplements

**FIGURE 1:**
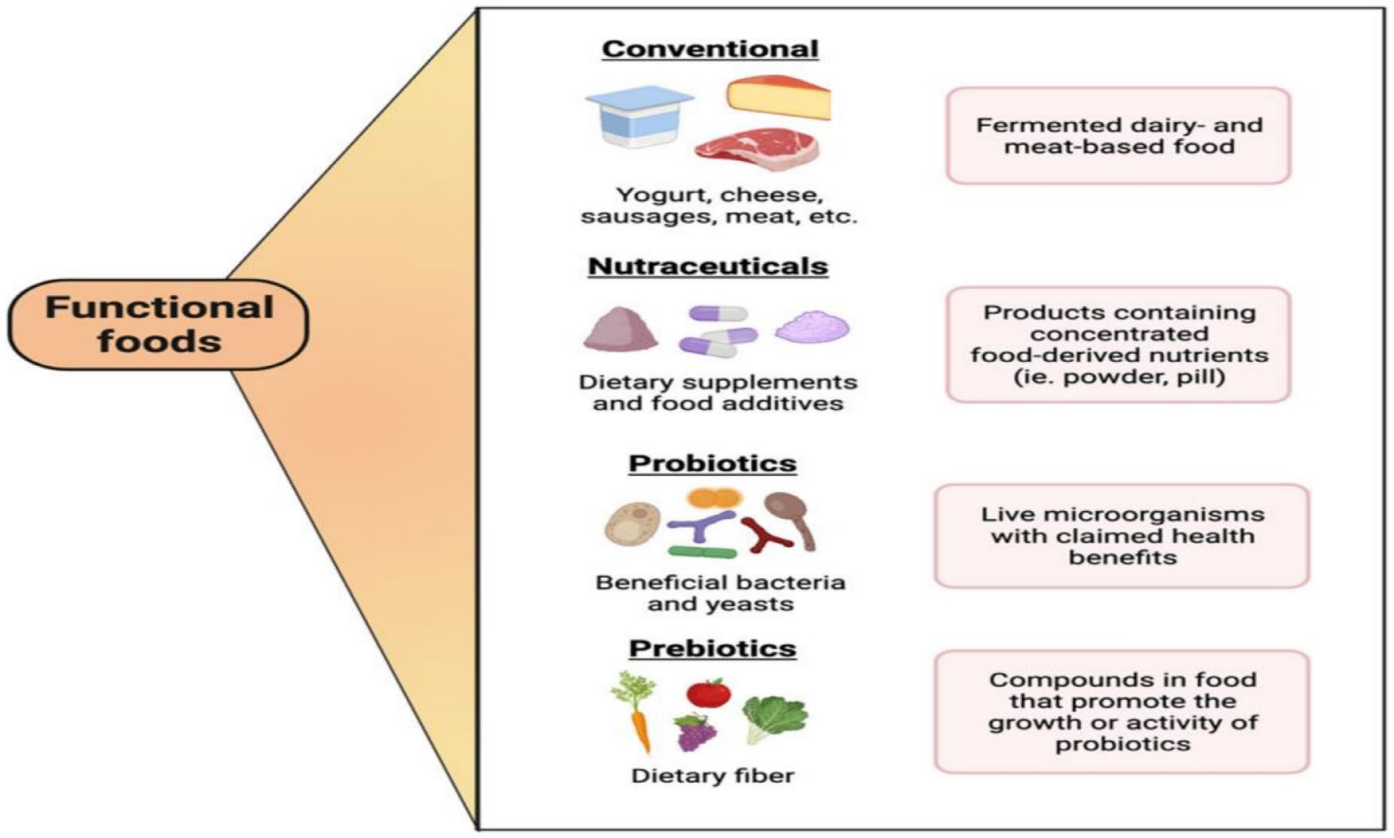
Functional foods using the Microbiome.

Consumer health and microbial science form an interesting crossroads where the latest developments in functional foods and supplements are being redefined. As research on the human microbiome has emerged, so too has the realization that altered microbiomes could influence the development of next generation probiotics and synbiotics. Probiotics have been defined as living bacteria that confer health benefits when administered in adequate amounts. Currently, the design of probiotics has increasingly focused on their specific roles within the human gut microbiome, targeting issues such as immune regulation and metabolic health (Pinu et al. 2019). Synbiotics are another form of microbial intervention in which probiotics are combined with prebiotics to enhance balance in gut ecosystems by fostering the growth of beneficial bacteria (Radaic and Kapila 2021). One such segment in which microbiome‐based functional foods are gaining momentum is metabolic health. Gut microbiota influence two essential functions of energy metabolism: fat accumulation and nutrient digestion (Pinu et al. 2019). Engineered probiotics are expected to reduce the risk of obesity, type 2 diabetes, and cardiovascular diseases by altering gut flora (Saleem and Saleem 2015). Numerous *Lactobacillus* and *Bifidobacterium* strains have been engineered to overproduce SCFAs, an outcome associated with altered insulin sensitivity and fat deposition (Papoutsoglou et al. 2023). These probiotic enhancements are currently under evaluation as part of the broader pursuit of integrating microbiome science into preventive health practices (Ru et al. 2020). Targeted probiotic therapies have been developed not only for metabolic health but also for the function of the microbiome in immunological regulation. The gut microbiota directly influences the immune system by modulating the function of immune cells such as T cells and macrophages, which are critical for reducing inflammation and infections (Paul et al. 2023). Engineered microbiomes are being produced to enhance this immune control and thus help the body fight infections while averting chronic inflammatory diseases (Muszer et al. 2015). It is vital in the context of allergies and autoimmune illnesses because the failure of the immune system has been related to the dysbiosis concept, which describes an imbalance in the microbial community (Satta et al. 2024). Another exciting field of study is the gut‐brain axis, where the microbiome is thought to play an essential role in mental health. Some bacterial types can synthesize neurotransmitters that affect mood and cognitive function, such as GABA and serotonin (Pinu et al. 2019). To manipulate the gut‐brain axis, microbiome‐based supplements are under investigation as potential treatments for various mental health disorders, including anxiety and depression (Sariola and Gilbert 2020). Indeed, certain strains, such as *Lactobacillus rhamnosus* , have been shown to reduce anxiety‐like behaviors in animal models and further emphasize the potential of personalized microbiomes for treating such mental health conditions (Figure 1) (Radaic and Kapila 2021). Figure 1 shows the functional foods using the microbiome.

### 7.2 Personalized Nutrition and Microbiome Modulation

Another revolutionary area in which microbiome research is making great strides is the emergence of personalized nutrition. The exact analysis of individual microbiomes, made possible by the development of metagenomics and other ‐omics technologies, can now be used to personalize nutritional treatments to maximize health outcomes (Schaerer et al. 2024). Personalized nutrition, as determined by gut microbiome composition, is said to include foods and nutrients that, by changing the gut microbiome, can achieve particular health outcomes with consideration of specific metabolic demands and susceptibilities (Petruschke et al. 2021; Rändler‐Kleine et al. 2020). Dietary regimens developed from such microbiome profiles can improve immunological responses and metabolic efficiency while reducing the incidence of chronic diseases (Olmo et al. 2022). Fiber‐rich dietary regimens will only enhance butyrate production in individuals with high populations of butyrate‐producing bacteria, further improving the output of this SCFA associated with its anti‐inflammatory properties (Salaheen et al. 2017). Similarly, diets high in antioxidants and omega‐3 fatty acids have anti‐inflammatory effects and may especially benefit individuals with a microbiome that favors pro‐inflammatory bacterial species (Nadarajah and Abdul Rahman 2023).

Recent research also shows that personalized nutrition may particularly benefit the management of chronic disorders, such as diabetes, cardiovascular disease, and IBD (Naeem and Selamoglu 2024). As a potential strategy in the management of blood sugar levels and improvement of insulin sensitivity in the context of diabetes, dietary therapies based on the microbiome are under investigation (Sessitsch et al. 2023); which involve the encouragement of the growth of beneficial gut microorganisms that impact glucose metabolism. Personalized diets that decrease dysbiosis and improve microbial diversity have also shown promise in reducing the symptoms of IBD and improving gut health in the long run (Newbold and Ramos‐Morales 2020; Saleem and Saleem 2015). Another potential role of tailored microbiome‐based nutrition is preventive health. Given the nature of these advances and the growing desire for more individuals to optimize their diet for lifelong health, profiling of the microbiome—a method through which further insight into disease prevention and overall well‐being can be gained—is likely in the near future to become a common feature of personalized medicine (Rimoldi et al. 2018). Indeed, the application of machine learning methodologies in microbiome studies extends the possibilities of predicting changes that individual microbiomes will undergo due to various dietary interventions, thus allowing for more precise and efficient nutritional recommendations (Sessitsch et al. 2023). In summary, the incorporation of microbiome science into functional food development and supplementation, in addition to personalized nutrition design, represents a great leap forward in the field of consumer health. As ongoing research elucidates increasingly complex connections between the microbiome and various health outcomes, there are opportunities for personalized microbial interventions within everyday diet and nutrition (Olmo et al. 2022). This could provide new avenues toward disease prevention and health optimization.

## 8 Ethical, Regulatory, and Social Implications in Microbiome Engineering

### 8.1 Navigating Regulatory Landscapes for Microbiome Innovations

The science of microbiome engineering is developing, and regulations are becoming increasingly challenging to navigate. Regulations involving microbiome‐based products differ depending on the area involved, and include probiotics, engineered synbiotics, and functional meals. Recently, regulations involving food products derived from the microbiome have been developed globally (Sidorova and Voronina 2020). For example, the FDA in the US regulates such products according to the category they fall into: food, dietary supplements, and medicinal agents. The European Food Safety Authority, for its part, calls for a stringent safety assessment of probiotics and other microbiome therapies before commercial distribution in the European Union. These differing standards reflect the need to balance innovation with consumer safety and signal some of the challenges faced by producers and researchers in this intricate environment (Sessitsch et al. 2023). Regulatory approval for products based on microbiomes necessitates proof of their efficacy and safety. Innovations such as the employment of genetically modified organisms in microbiome engineering raise other ethical and regulatory issues. For example, there is great potential to use genetically modified probiotics to enhance health outcomes. However, unintended and unseen effects may include disturbances in native microbial communities and horizontal gene transfer (Tsiknia et al. 2021). Consequently, regulatory mechanisms are now concerned with addressing the consequences of these advances with regard to environmental and long‐term safety along with their health benefits (Stoddard et al. 2024). The second challenge is the ethical issue that arises from microbiome interventions. The most common ethical concerns pertain to consumer safety, environmental sustainability, and label transparency (Walker and Buhler 2020). Since changed microbiomes enter the food chain, there is an imminent need for legislation that guarantees assurance to customers about what exactly they consume and how safe the product is (Thakur et al. 2023). This includes clear labeling of the components in food that are either genetically modified or altered by microbiota, so that the customer can make a choice based on their judgment. There needs to be an ethical sense of responsibility when advocating microbiome engineering innovation to win over the public for commercial success (Von Braun et al. 2021).

### 8.2 Consumer Perceptions and Market Dynamics

Despite the possible health benefits, customers are generally wary of buying products derived from improved microbiomes. The concept that genetically modified organisms are unnatural is one of the greatest causes of concern for most people when introduced into food systems (Vujanovic et al. 2022). Research has indicated that customers could be skeptical of products involving microbiome engineering owing to the fear of unpredictable results on their gut microbiome or causing long‐term health problems (Wassermann et al. 2022). These are matters that open dialogue and education can address. Clear communication about the benefits and safety of products in evidence‐based information will enhance consumer knowledge and acceptability of microbiome‐engineered goods (Singh and Trivedi 2017). This may be facilitated through public health campaigns, education labeling, and the inclusion of customers in discussions on microbiome science. Knowing how altered microbiomes can facilitate metabolic function, strengthen the immune system, and improve nutrient uptake, all factors contributing to health may allay consumer fears and build a better rate of acceptance (Topçuoğlu et al. 2020). The development of microbiome‐related innovations is also heavily influenced by market forces (Von Braun et al. 2023). Although the market for microbiome‐based products and personalized nutrition is developing, consumers are increasingly health‐oriented, and they trust the science underpinning microbiome engineering. Regulatory regimes that ensure product safety are crucial for the success of these products (Wiesmann et al. 2023). To cultivate such confidence, companies would have to be candid about what microbiome engineering is, what risks it carries, and the measures taken to ameliorate these risks (Yoon et al. 2021). Clever marketing emphasizing the environmental friendliness and health benefits of microbiome‐based products could help place such developments within the context of a greater trend toward personalized, health‐enhancing food products (Zhan et al. 2022). In summary, the social, legal, and ethical implications of microbiome engineering are multi‐layered and continuously changing. Regulations need to move along with the rapid strides that research is underway in microbiome science without losing the confidence and safety of consumers (Rodriguez et al. 2024). Companies developing products related to the microbiome will have to engage consumers through transparency, business ethics, and education to overcome skepticism and achieve market acceptance. When overcome, these challenges will unlock the full potential of the microbiome in food systems and beyond, as they continue to emerge with a critical role in consumer health (Zhou and Gallins 2019).

## 9 Research Opportunities

In the future, microbiome studies will be more interdependent, from data science through bioinformatic food science to microbiology. Such an approach is necessary to further understand microbiome technology and apply it. The development of novel, safe, and efficacious probiotic and synbiotic products for humans will be achieved through the interplay between food science and microbiology (Sessitsch et al. 2023). Microbiologists and food scientists can work together to improve the number of strains that enter food products and deliver a viable number through the digestive system to elicit their health benefits. However, bioinformaticians do not fall behind in this endeavor, as they are hugely engaged in deciphering the complex data generated in the study of the microbiome. By applying sophisticated computational methods, one can certainly identify patterns and associations within microbiome data that may not be clearly visible using more conventional approaches (Sidorova and Voronina 2020). Data science approaches, through predictive modeling and analysis techniques, are revolutionizing microbiome research on interactions with microbiome hosts using artificial intelligence and machine learning (Singh and Trivedi 2017). Such technologies analyze large volumes of data to predict the possible implications of different microbiome compositions on food quality and health outcomes. Thus, a combination of machine learning models with multi‐omics data—genomics, proteomics, and metabolomics—can advance our understanding of microbiome functions and predict the outcomes of microbiome interventions on human health (Topçuoğlu et al. 2020). Predictive microbiome management will be able to predict how changes in the microbiome will affect food systems and human health, which will be driven by computational models and simulations capable of predicting the influence of different interventions on composition and function. Such a prediction capability can also enable personalized nutrition strategies based on unique microbiome profiles that may yield optimized health outcomes (Trif et al. 2022). Synthetic biology is another pathway for microbiome engineering innovation. This will enable the production of desired microbiomes with specific functions by designing new biological parts, technologies, and systems (Trivedi et al. 2021). This strategy would more than likely lead to new probiotics that provide improved health benefits, or to the development of treatments for chronic diseases based on microbiomes. For example, it would be possible to engineer microbes capable of producing bioactive compounds or degrading environmental pollutants at a higher rate (Tsiknia et al. 2021).

### 9.1 Emerging Technologies and Innovative Approaches

However, even as CRISPR/Cas9 has taken genetic engineering into a novel era, the pursuit of competing editing techniques that could possess some singular advantages is gathering steam. Technologies such as prime editing and base editing enable finer mesh control over genetic changes without inducing double‐strand breaks. These more sophisticated technologies can, for instance, confer specific traits to microbiomes to greatly improve their tolerance to extreme environmental conditions or enhance their synthesis of compounds of interest (Von Braun et al. 2023). In addition to genetic editing, new methods for manipulating the microbiome, such as phage treatment and antimicrobial peptides, have been explored for their potential to reshape microbial communities (Von Braun et al. 2021). In some instances, such alternative methods may have greater efficacy than traditional genetic tools and thus offer new perspectives on how microbiomes can be managed within food systems and elsewhere. The development of effective delivery methods is essential if microbiome‐based therapies are to be successful clinically or on farms. Innovations in delivery systems, such as hydrogels and microencapsulation, have been used to enhance the survivability and efficacy of probiotics and other microbiome‐based products (Vujanovic et al. 2022).

Microencapsulation promotes survivability by processing and storage while protecting microorganisms from environmental challenges. Hydrogels, on the other hand, may be employed in the delivery of probiotics into the gastrointestinal system in a controlled fashion, thus ensuring the active delivery of nutrients to appropriate sites where their action is desired (Walker and Buhler 2020). Recent developments in nanotechnology have resulted in more advanced delivery vehicles. Probiotics and other drugs used in microbiome modulation can be encapsulated in nanosized carriers, which would realize higher bioavailability in circulation and thus be distributed at specific sites and in greater amounts. These novel approaches are opening new opportunities toward more targeted and effective treatments that might result in improved health outcomes and enhanced product utility based on the microbiome (Wang, Wang, Xiao, et al. 2024). Interdisciplinary collaboration and technological development will also greatly benefit future microbiome research. By integrating knowledge from many disciplines and investigating the use of new technologies, it will be possible for researchers to open up entirely new avenues for the manipulation and exploitation of microbiomes (Wang et al. 2019). The improvement in the tools and methods of delivery in microbiome editing is promising for solving current problems and creating new opportunities for innovation in microbiome science (Wang et al. 2019). These aspects will play a fundamental role in establishing the basis for how the engineering of microbiomes will continue to develop in the future and how they are applied in agriculture and health (Wang, Wang, Xiao, et al. 2024).

## 10 Conclusion and Future Perspectives

In conclusion, it is emphasized that the primary scope of this review is anchored in food science, with food systems providing a broader context and nutrition and health serving as indirect, consequential dimensions. Studies on microbiomes have revolutionized our understanding of food science by highlighting the complex ways in which microbial communities interact to affect all aspects of food production and consumption. Microbiomes are crucial in the development of sustainable food systems, given their effect on food safety and nutrition, as well as their potential for enhancing crop health and soil fertility. This review focuses on the ecological connections between microbiomes and their hosts and how such relationships may be manipulated to improve agricultural practices and food quality. Technological advances that are being realized in precision microbiome engineering provide exciting prospects for the optimization of food systems. By applying state‐of‐the‐art delivery technologies, CRISPR‐based editing, and synthetic biology, researchers are generating bespoke microbiome interventions targeting specific problems in food production and health. In addition, these may enhance nutritional profiles, develop functional foods, and cure chronic diseases using personalized nutrition strategies. However, these developments have raised social, legal, and ethical issues that affect the translation of microbiome science to applications within food systems. What is urgently needed is a balanced approach that favors innovation, but at the same time guarantees consumer safety. Understanding consumer attitudes and market conditions is critical for the successful implementation of microbiome‐driven technologies. Yet, as we forge ahead, we must bear in mind the far‐reaching implications of microbiome technologies and their potential to contribute to more resilient and sustainable food systems. Most importantly, microbiome innovation must reconcile high‐tech advancement with low‐tech location‐specific practices. For the majority of LMIC food systems, low‐tech approaches such as natural fermentation, crop–soil microbiome symbiosis, and dietary diversity are the most practical and culturally acceptable. Ensuring that microbiome science engages in precision engineering and indigenous knowledge is crucial to equity, practicality, and reach.

Let me conclude by saying that the power of microbiomes can revolutionize food science. In this process, embracing the complexity of microbiological interactions and moving forward with precision engineering methods, a new response can be created to the problems presented by the rapidly changing world. Several disciplines, such as food science, microbiology, bioinformatics, and data science, are likely to converge in upcoming microbiome investigations in food science. This will stimulate interdisciplinary collaboration that could advance predictive microbiome management and novel genome‐editing platforms. In addition to CRISPR, advances in the field of microbiome‐editing technologies have the potential to increase the accuracy and efficiency of microbiome interventions and possibly change the nature of food production and processing. Newer delivery methods, such as hydrogels and microencapsulation, will enhance the stability and efficacy of microbiome‐based solicitations in food. Further progress in these technologies holds strong potential to build safer, healthier, and more sustainable food systems that address critical challenges in public health, environmental sustainability, and food security. While microbiome technologies hold promise, they also bring with them potential risks and trade‐offs, including ecological imbalances, regulatory entry obstacles, and socio‐economic inequalities. What may be feasible for industrialized food systems in high‐income economies may not be directly transferable to smallholder producers in LMICs. Therefore, microbiome‐based interventions must be context‐specific, integrate high‐tech and low‐cost traditional technology, and be supported by robust policy and regulatory environments.

## Author Contributions

**Muhammad Tayyab Arshad:** conceptualization (equal), data curation (equal), methodology (equal), validation (equal), visualization (equal), writing – original draft (equal). **Sammra Maqsood:** data curation (equal), investigation (equal), methodology (equal), visualization (equal), writing – original draft (equal). **Md. Sakhawot Hossain:** data curation (equal), methodology (equal), software (equal), visualization (equal), writing – review and editing (equal). **Farhang Hameed Awlqadr:** validation (equal), writing – review and editing (equal). **Abdul Rauf:** data curation (equal), resources (equal), writing – review and editing (equal). **Iffat Ullah:** data curation (equal), formal analysis (equal), writing – review and editing (equal). **Ali Ikram:** conceptualization (equal), project administration (equal), validation (equal), writing – review and editing (equal). **Ayesha Bibi:** resources (equal), writing – review and editing (equal). **Sayeed Mukhtar:** validation (equal), writing – review and editing (equal). **Muhammed Adem Abdullahi:** conceptualization (equal), writing – review and editing (equal).

## Funding

The authors have nothing to report.

## Disclosure

The authors have nothing to report.

## Ethics Statement

The authors have nothing to report.

## Consent

The authors have nothing to report.

## Conflicts of Interest

The authors declare no conflicts of interest.

## Data Availability

The data that support the findings of this study are available from the corresponding author upon reasonable request.
